# Cancer Incidence and Mortality Through 2020

**DOI:** 10.5888/pcd13.160024

**Published:** 2016-04-07

**Authors:** Hannah K. Weir, Mary C White

**Affiliations:** Author Affiliation: Mary C. White, Centers for Disease Control and Prevention, Atlanta, Georgia.

**Figure:**
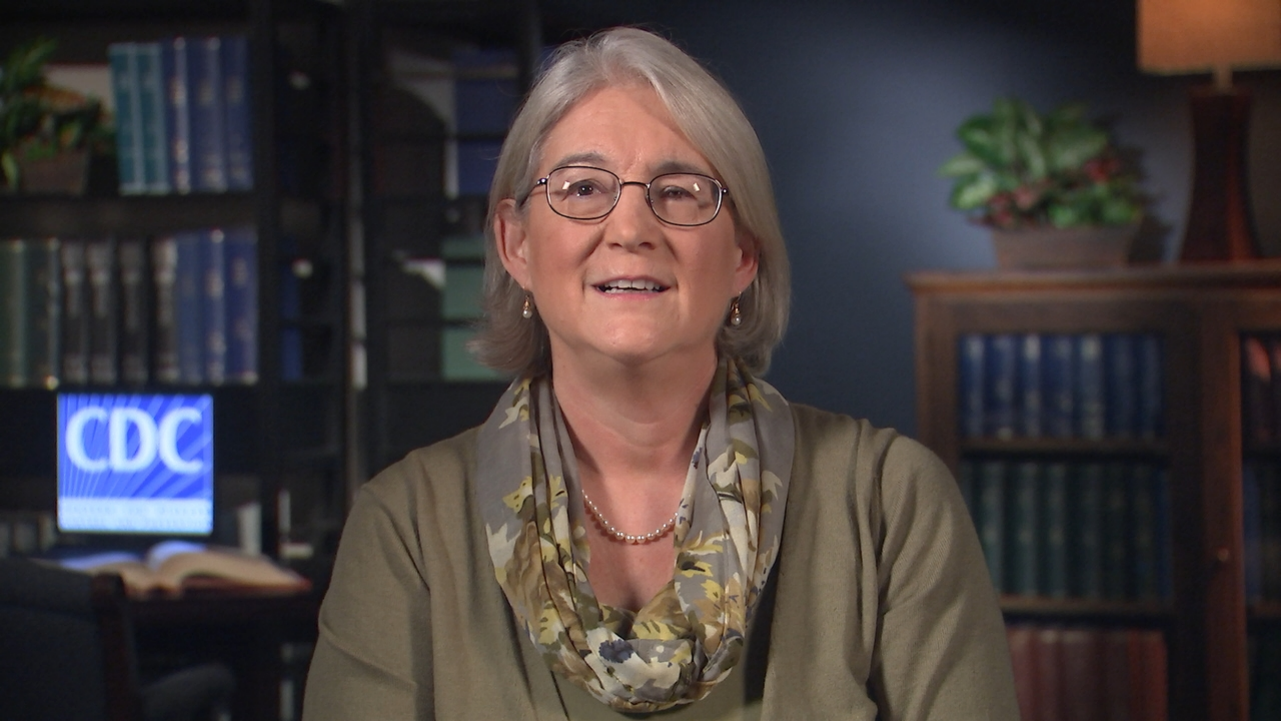
Centers for Disease Control and Prevention epidemiologist Dr Hannah Weir talks about her work to predict cancer incidence and mortality through 2020. Her predictions are based on population projections from the US Census Bureau and on national cancer surveillance data from the National Program of Cancer Registries and the National Vital Statistics System. Projections were calculated by using age–period–cohort regression models. Although cancer rates are decreasing or stabilizing, cancer incidence and mortality will continue to rise (1,2). The public health community needs to do more to address these increases by reducing the number of people who get cancer through prevention and by reducing the number of people who die of cancer through early detection and treatment. Results of this work were previously published in both *Preventing Chronic Disease* and *Cancer.* Run time: 05:12. https://www.youtube.com/watch?v=MJp4IfboItw

## References

[1] Weir HK , Thompson TD , Soman A , Møller B , Leadbetter S , White MC . Meeting the Healthy People 2020 objectives to reduce cancer mortality. Prev Chronic Dis 2015;12:E104. 10.5888/pcd12.140482 26133647 PMC4492213

[2] Weir HK , Thompson TD , Soman A , Møller B , Leadbetter S . The past, present, and future of cancer incidence in the United States: 1975 through 2020. Cancer 2015;121(11):1827–37. 10.1002/cncr.29258 25649671 PMC4507799

